# Supplementary material for: Phototaxis beyond turning: persistent accumulation and response acclimation of the microalga Chlamydomonas reinhardtii
Source: Sci Rep. 2017 Jun 14;7:3447. doi: 10.1038/s41598-017-03618-8 (PMC5471259; doi:10.1038/s41598-017-03618-8)
Supplement: Supplementary file 2 — Supplementary Informations [file 41598_2017_3618_MOESM2_ESM.pdf]

Phototaxis beyond turning: persistent accumulation and response acclimation of the microalga *Chlamydomonas reinhardtii*.
Supplementary Informations.

Jorge Arrieta[1], Ana Barreira[1], Maurizio Chioccioli[2], Marco Polin[3*], and Idan Tuval[1*]
[1] *Mediterranean Institute for Advanced Studies (CSIC-UIB), Spain*
[2] *Cavendish Laboratory, University of Cambridge, Cambridge CB3 0HE, United Kingdom*
[3] *Physics Department, University of Warwick, Gibbet Hill Road, Coventry CV4 7AL, United Kingdom*
[*] *Correspondence to M.P. (email: m.polin@warwick.ac.uk) or I.T. (email: ituval@imedea.uib-csic.es).*

Supplementary movie

The movie Mov1s.avi shows the dynamics of cell accumulation and dispersion in our experiments for a single light on - light off cycle.

Models for single cell phototaxis

The position $\mathbf{x}(t)$ of a cell swimming at constant speed v_s along the direction $\mathbf{p}(t)$ will evolve according to

$$\dot{\mathbf{x}}(t) = v_s\mathbf{p}(t) \quad ; \quad \dot{\mathbf{p}}(t) = \boldsymbol{\omega} \wedge \mathbf{p}(t), \tag{1}$$

where the angular speed $\boldsymbol{\omega}$ encodes the phototactic response through its (unknown) dependence on the light field. Absent detailed measurements, a common approach has been to assume proportionality to the local gradient in light intensity, $\boldsymbol{\omega} = \alpha\mathbf{p}(t) \wedge \nabla I$, where the phototactic parameter α, possibly dependent on I, represents the magnitude of the response.

The model used throughout the manuscript uses the simplest description that incorporates $\alpha(I) =$ constant. Here we address two additional functional forms for $\alpha(I)$ chosen to model (a) the transition from positive to negative phototaxis and (b) the transition from positive to dia-phototaxis. For the former, we chose a continuous function of I that changes sign at a prescribed critical intensity; more specifically, we set $\alpha(I) = 1 - 2\exp(I/I_c)$ as seen in Fig. S1a (blue curve). An example of a trajectory corresponding to this choice of $\alpha(I)$ is depicted with the same colour in panel (b). For the model to incorporate a transition to diaphototaxis it is necessary to include in the equation for $\boldsymbol{\omega}$ a term proportional to $\mathbf{p}(t) \cdot \nabla I$. In our case cells are confined to move on the xy plane and therefore $\boldsymbol{\omega} = \omega\hat{e}_z$ where $\hat{e}_z$ is the unit vector in the direction perpendicular to the plane of motion. We have explored as a particular realisation of the phenomenological diaphototactic model the following:

$$\omega = \alpha(I)(\mathbf{p}(t) \wedge \nabla I) \cdot \hat{e}_z + (1 - \alpha(I))\mathbf{p}(t) \cdot \nabla I, \tag{2}$$

with $\alpha(I) = \exp(I/I_c)$. The model represents a continuous decay of positive phototaxis to zero for $I > I_c$ (shown as a magenta line in Fig. S1a) and a concurrent increase of the diaphototactic contribution (dashed magenta line in the same figure). The combined effect of the two contributions leads to the cell circling around the position of $I = I_c$ as shown for the trajectory plotted with the same colour in panel (b).

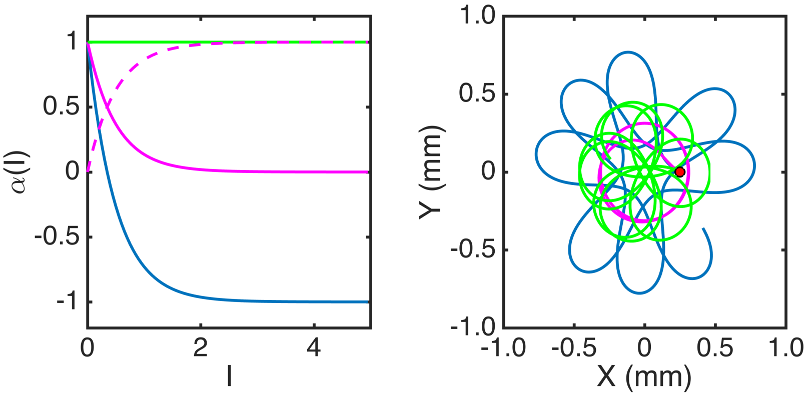

FIG. S1. $\alpha(I)$ (a) and representative trajectories (b) for the three different individual based models explored. All the trajectories were initialized starting at $\rho = \rho_c$ with initial orientations $\theta = 205°$. The local gradient model used throughout the manuscript (green) as compared with a model that includes a transition from positive to negative phototaxis at a critical light intensity I_c (blue) and with a phenomenological model incorporating diaphototaxis (magenta). The critical light intensity was set to $I_c = 0.5$ in all cases.

Dynamics of mixed-population response

To assess if the reduced phototactic sensitivity measure at the population level is not a consequence of mixed single-cell responses (positive or negative taxis) present at different ratios as a function of light exposure, we performed further

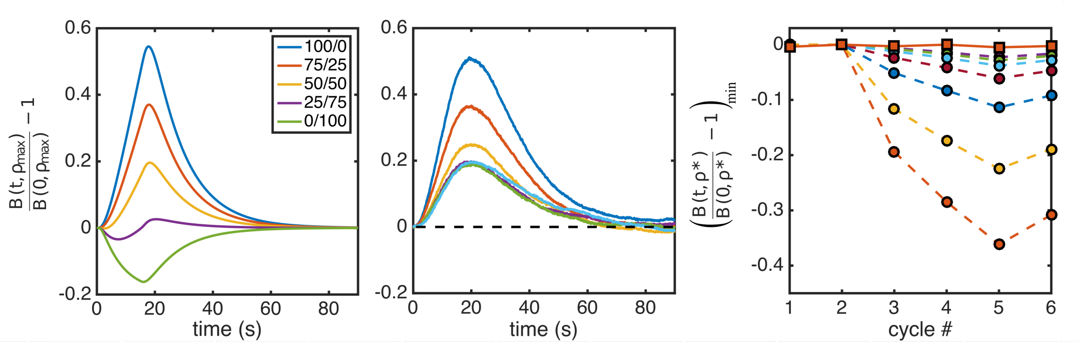

FIG. S2. Comparison between mixed-population and experimental behaviour. a) Accumulation curves at $\rho = \rho_{max}$ during one on/off cycle for varying proportions of positive/negative cells; b) Representative experimental accumulation curves at $\rho = \rho_{max}$ for six successive on/off cycles (blue; red; orange cyan; purple; green); c) Circles: simulated minimum of the normalised accumulation curves through six on/off cycles, for mixed-response populations corresponding to the experimental values of β shown in Fig. 3(b) for 7 values of $\rho* = [0.4, 0.5, 0.6, 0.7, 0.8, 0.9, 1.0]\rho_{max}$; Squares: experimental minima for the normalised accumulation curves for $\rho = 383\mu m$ through six on/off cycles.

tests on our experimental data which strongly support our original interpretation.

If we imagine to look at a population of cells phototaxing within e.g. a straight channel, a population of reduced response and one of mixed response can give exactly the same net current of cells and therefore be indistinguishable, at least from a macroscopic, continuum perspective. However, this parallel does not always hold. For example, the two cases are different at boundaries like the beginning and end points of the channel. For mixed-response populations, these regions will deplete of one type of cell and increase in the other, and as a result the total number of cells in a region close to the boundary will in general display an initial dynamics which is qualitatively different from the case of a single population with reduced response. This happens during the time required to empty the observation region of the cells of the type that is accumulated at the other end. In particular, this initial transient can include a decrease in cell concentration, rather than the constant increase seen in a homogeneous population. This is the case for the population experiments we have performed.

In our case, the role of the boundary is played by the centre of the light field, the region which the negatively phototacting cells in the mixed population would escape from. We used the experimentally-validated population model in the paper to simulate the behaviour of mixed-response populations for increasingly larger fractions of negatively phototacting cells. Fig. S2(a) shows the resulting (normalised) accumulation curves $B(t, \rho)$, for $\rho = 958\mu m$ (ρ_{max}) and increasing proportion of negatively phototacting cells. The curves show a clear decrease above a critical proportion of negatively phototaxing cells, followed by a minimum and a final increase (as long as the percentage of positively phototaxing cells is above 25%).

Considering the linear increase part in the normalised $B(t, \rho_{max})$ we estimated which proportions of negatively phototacting cells would correspond to the values of β we observed through the cycles (see Fig. 3(b) of the main manuscript). These were then used to simulate the evolution of the concentration profiles through the cycles, in case of a mixed-response population.

The mixed-response profiles show accumulation dynamics that are qualitatively different from those observed experimentally. In particular, they display an initial minimum whose magnitude increases through the cycles. Figure S2(c) (circles) shows how these minima evolve as the cycles progress, for 7 values of $\rho*$ between $383\mu m$ and $958\mu m$ ($0.4\rho_{max} < \rho < \rho_{max}$). This should be compared with the minima of the experimental accumulation curves (squares), which are shown here for $\rho = 383\mu m$ (the value providing the deepest minima for the mixed-response population). Experimental curves for the other values of ρ are equivalent.

These plots show that, in experiments, turning on the light determines an increase in the number of cells within any circular area around the centre, and for any cycle number. The experiments have a clear qualitative difference from the behaviour that would be expected from a mixed-response population, and therefore are not compatible with the hypothesis that the adaptation is due to a varying proportion of negatively vs. positively phototaxing cells.

Strain choice for chlorophyll autofluorescence

Our current set-up for measuring fluorescence requires to have a constant number of cells in the field of view for long enough to correctly assess the long-timescale acclimation dynamics (tens of minutes), while avoiding changes in the fluorescence signal coming from changes in local cell concentration. This makes working directly with motile CC125 cells challenging. One possibility is to use de-flagellated CC125 cells, but this presents two major problems. Firstly, flagellar regrowth happens on a time-scale similar to the one we are exploring here, thus triggering partial cell motility which can interfere with our measurements. Secondly, de-flagellation impacts cell metabolism, and therefore this might have affected the results in an unpredictable way. Using an immotile mutant is a way to circumvent both limitations. CC2905 cultures were grown with the same protocol that we used for CC125 cells, under constant agitation in an orbital shaker. The cultures were always kept at mid-log phase and showed no sign of cell aggregation.
